# Unveiling Mycobacterium fortuitum Lung Disease: A Case Linked to Pedicure Practices

**DOI:** 10.7759/cureus.84758

**Published:** 2025-05-24

**Authors:** Ivana Y Mangione Leon, Losan Alashi, Gerardo Munoz Monaco, Carolina Gomez de Ziegler

**Affiliations:** 1 Family Medicine, University of Texas Rio Grande Valley, Edinburg, USA; 2 Family Medicine, University of Texas Rio Grande Valley, Weslaco, USA; 3 Family Medicine, Knapp Medical Center, University of Texas Rio Grande Valley, Weslaco, USA

**Keywords:** clinical case report, lung disease, mycobacterium fortuitum, pedicure infection, rare cause of infection

## Abstract

*Mycobacterium fortuitum* (*M. fortuitum*) is a rapidly-growing mycobacterium (RGM) that can lead to a variety of infections affecting the lungs, skin, and soft tissue. Pulmonary infections with *M. fortuitum* primarily affect individuals with pre-existing lung conditions, although exposure through contaminated water sources, such as pedicures and nail salon whirlpool footbaths, has also been documented. We present a case of a previously healthy young male presenting with respiratory symptoms and systemic signs suggestive of infection. Following extensive diagnostic workup, *M. fortuitum* was identified in sputum samples, leading to a favorable response to targeted antibiotic therapy. This case underscores the importance of considering *M. fortuitum* in differential diagnoses of pulmonary infections and skin infections, particularly in patients without known risk factors.

## Introduction

*Mycobacterium fortuitum* (*M. fortuitum*) is a rapidly-growing mycobacterium (RGM) often found in the environment, which can lead to pulmonary disease and skin and soft tissue infections. When it presents as a pulmonary infection, it frequently affects individuals with existing lung conditions such as pulmonary tuberculosis, interstitial lung disease, or lung cancer. Additionally, it can be related to exposure to pedicures and nail salon whirlpool footbaths [1,2]. Although *M. fortuitum* is frequently detected in respiratory specimens, relatively few cases of pulmonary infections require antibiotic treatment, and when treatment is needed, a positive outcome can be achieved with the right antibiotics [2].

## Case presentation

We present the case of a 20-year-old male patient with an unremarkable past medical history who presented to the emergency department with one month of progressive cough associated with shortness of breath, fever, bilateral testicular pain, and weight loss. The patient had no significant exposures, sick contacts, or recent travels. He only reported going to a nail salon for a pedicure two weeks before symptoms developed.

Vital signs on admission were temperature of 100°F, blood pressure of 112/76 mmHg, heart rate of 115 beats per minute, respiratory rate of 20 breaths per minute, and oxygen saturation of 98% on room air. Physical examination revealed a distress-appearing, alert patient with dry mucous membranes, anterior and posterior cervical lymphadenopathy, tachycardia with regular heart rhythm, rales and wheezing, and bilateral inguinal lymphadenopathy.

Laboratory findings included white blood cell count of 23.73 x 10^9/L, hemoglobin of 14.5 g/dL, platelets of 559 x 10^9/L, creatinine of 1.09 mg/dL, glucose of 158 mg/dL, aspartate aminotransferase (AST) of 20 U/L, alanine aminotransferase (ALT) of 23 U/L, lactic acid of 1.99 mmol/L, thyroid-stimulating hormone (TSH) of 0.14 mIU/L, and urinalysis negative.

The patient was admitted with sepsis secondary to respiratory tract infection. Sepsis protocol was followed, and the patient was started on vancomycin and piperacillin/tazobactam. A multidisciplinary team, including pulmonology and infectious disease specialists, was consulted. Chest X-ray reported fluffy confluent densities on bilateral bases with increased interstitial markings, most likely an acute infectious inflammatory process. CT of the chest reported multiple nodular densities (tree in-bud) on the anterior segments of the upper lobe, middle lobe, lingular segment on the left side, and basal segments bilateral, demonstrating chronic granulomatous pleuro-pulmonary infection. Ultrasound of bilateral testis reported bilateral epididymitis. During hospitalization, infectious diseases recommended adding doxycycline to the antibiotic regimen. A bronchoscopy was performed as per the pulmonologist's recommendation. Work-up including Histoplasma antigen, acid-fast bacillus (AFB) culture and smear, Aspergillus antibody, mumps IgM, cryptococcus antigen, Coccidioides antibodies, urine chlamydia/GC, antinuclear antibody (ANA), antineutrophil cytoplasmic antibodies (ANCA), cytomegalovirus (CMV), toxoplasma, QuantiFERON, blood cultures, respiratory panel, HIV, rapid plasma reagin (RPR), Legionella, and purified protein derivative (PPD) was negative. The patient tested positive for *M. fortuitum* on AFB culture from induced sputum and bronchial washings (Table 1).

**Table 1:** AFB culture and susceptibilities from bronchial washings. Growth of *Mycobacterium fortuitum* (*M. fortuitum*) complex from induced sputum and bronchial washings. AFB: acid-fast bacillus.

Susceptibility *M. fortuitum*	Minimum inhibitory concentration	Results
Amikacin	<1	Susceptible
Cefoxitin	<16	Susceptible
Ciprofloxacin	<0.5	Susceptible
Clarithromycin	1.0	Susceptible
Clofazamine	0.25	Susceptible
Doxycycline	<0.12	Susceptible
Imipenem	2.0	Susceptible
Linezolid	2.0	Susceptible
Moxifloxacin	0.12	Susceptible
Tigecycline	0.12	Susceptible

**Figure 1:**
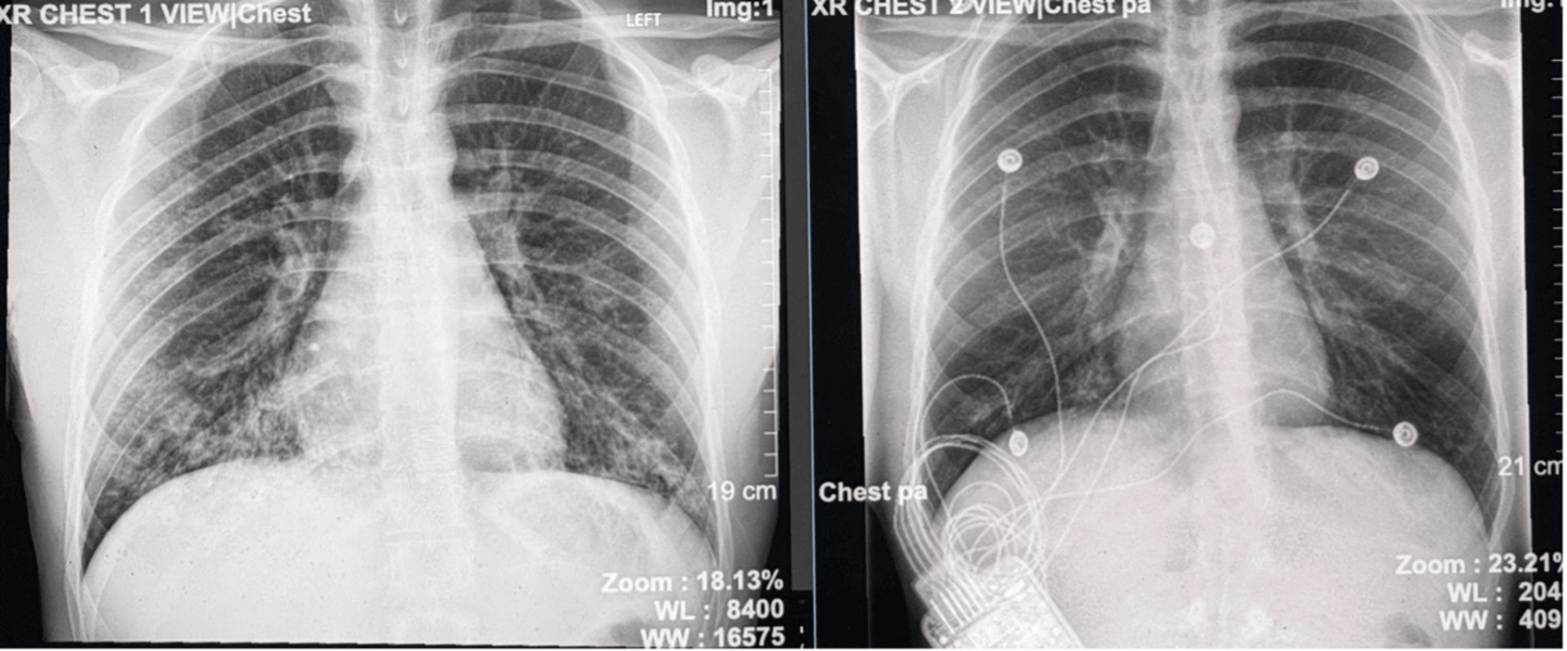
Chest X-ray images. A. Chest X-ray on admission. B. Chest X-ray prior to discharge.

The patient demonstrated clinical improvement and remained hemodynamically stable. A follow-up chest radiograph obtained prior to discharge revealed clear lung fields with no focal infiltrates. When compared to the initial imaging performed at the time of admission, there was near-complete resolution of the previously noted bibasilar infiltrates. No new or acute radiographic abnormalities were identified, and there were no findings suggestive of ongoing or emerging pathology (Figure 1). The patient was discharged home with doxycycline to complete 14 days of treatment and follow-up with a primary care physician.

## Discussion

*M. fortuitum* is a rapidly growing mycobacterium commonly associated with environmental sources, including water and soil. Diagnosing RGM infections can be challenging due to the broad variability in clinical presentation of skin lesions. This pathogen can cause various infections, particularly following medical or cosmetic procedures, such as pedicures in whirlpool footbaths. Primary cutaneous infections may emerge weeks to months after exposure, often resulting from direct inoculation via trauma, injections, or contaminated surgical instruments [2-3]. Previous outbreaks linked to nail salons have raised awareness about the potential for *M. fortuitum* to cause community-acquired infections. While respiratory infections with *M. fortuitum* are less common, they tend to affect individuals with underlying lung disease or immunocompromised states.

In October 2000, a study investigated an outbreak of *M. fortuitum* skin infections linked to whirlpool footbaths at a nail salon in northern California, where over 100 pedicure clients developed persistent, scar-forming boils on their lower legs [2]. Sampling from 10 footbaths revealed *M. fortuitum* in each unit, with identical strains in three footbaths and 14 patients, highlighting the need for stringent sanitation protocols in salon environments [2].

In 2002, Winthrop et al. reported a significant outbreak of community-acquired infections involving rapidly growing mycobacteria [2]. Over 115 patrons from a single nail salon developed severe lower-extremity *M. fortuitum* furunculosis due to contaminated whirlpool footbaths used in the salon. Since then, additional outbreaks and sporadic cases have revealed that mycobacterial infections linked to whirlpool footbaths are more common than previously thought. Because these infections have only recently come to light, their natural progression and best clinical management practices still need to be better defined [2].

The American Thoracic Society (ATS) 2007 guidelines are designed for other types of non-tuberculous mycobacterial pulmonary disease (NTM-PD), like those caused by *Mycobacterium avium* complex or *Mycobacterium abscessus*, and there is limited information on the best antibiotic treatments and prognosis for *M. fortuitum *[4]. According to the 2007 guidelines from the ATS and the Infectious Diseases Society of America (IDSA), *M. fortuitum* is highly susceptible to amikacin, ciprofloxacin, ofloxacin, sulfonamides, and imipenem, with varying susceptibility to cefoxitin, clarithromycin, and doxycycline [4]. Previous case reports have indicated that treatment combining antibiotics, including quinolones, has been successful [5]. In this case, the patient responded favorably to a regimen that included doxycycline, underscoring the importance of selecting appropriate antibiotics based on susceptibility testing.

## Conclusions

This case highlights the need to consider *M. fortuitum* as a potential respiratory pathogen, even in previously healthy individuals without significant exposure history or in patients exposed to nail salons and whirlpool footbaths. Effective management of *M. fortuitum* infections relies on prompt recognition, thorough diagnostic workup, and tailored antimicrobial therapy. Empiric treatment should be informed by local resistance data and adapted based on susceptibility testing results to optimize patient outcomes and prevent the overuse of broad-spectrum antibiotics. Further research is needed to establish more comprehensive treatment guidelines for *M. fortuitum* and other rapidly growing mycobacterial infections.
